# Efficacy and safety of wait and see strategy versus radical surgery and local excision for rectal cancer with cCR response after neoadjuvant chemoradiotherapy: a meta-analysis

**DOI:** 10.1186/s12957-020-02003-6

**Published:** 2020-08-31

**Authors:** Guo-hua Zhao, Li Deng, Dong-man Ye, Wen-hui Wang, Yan Yan, Tao Yu

**Affiliations:** 1 Department of General surgery, Cancer Hospital of China Medical University, Liaoning Cancer Hospital & Institute, No 44 Xiaoheyan Road, Dadong District, Shenyang, 110042 Liaoning Province PR China; 2 The Affiliated Hospital of Liaoning University of traditional Chinese Medicine, No 33 Beiling Street, Huanggu District, Shenyang, 110042 Liaoning Province PR China; 3 Department of Medical Imaging, Cancer Hospital of China Medical University, Liaoning Cancer Hospital & Institute, No 44 Xiaoheyan Road, Dadong District, Shenyang, 110042 Liaoning Province PR China

**Keywords:** Wait and see, Radical surgery, Local excision, cCR, Meta-analysis

## Abstract

**Background:**

Neoadjuvant therapy can shrink tumors, increase anus preservation rate, and protect anal function. Radical surgery need cut off the diseased bowel, clean up the lymph nodes, and then restore bowel function. It could bring traumatic effect and poor postoperative quality of life to the patient. Local resection requires removal of the diseased bowel with circular negative margin. The surgical trauma is small, and the postoperative quality of life is good. In this meta-analysis, we aimed to evaluate the efficacy and safety between wait and see strategy (WS), radical surgery (RS), and local excision (LE) of rectal cancer patients with clinical complete response (cCR) response after neoadjuvant chemoradiotherapy.

**Methods:**

We searched PubMed, Cochrane Library, CNKI (China National Knowledge Infrastructure), and Wanfang databases to compare wait and see strategy with radical surgery and local excision for rectal cancer with cCR response after neoadjuvant chemoradiotherapy up to March 2020. We collected the data of local recurrence, distant metastasis, cancer-related death, overall survival, and disease-free survival and used RevMan 5.0 to carry out the meta-analysis. Continuous data were evaluated by the standardized mean differences (SMD) with 95% confidence intervals (95% CIs), and dichotomous data were evaluated by relative risks (ORs or RRs) with 95% CIs. We aimed to compare the advantages and disadvantages of the three groups.

**Results:**

Eleven English studies with 1131 patients were included. There were 412 patients in WS group, 678 patients in RS group, and 41 patients in LE group. WS group had a higher local recurrence rate than RS group (OR 7.32, 95% CI 3.58 to 14.95, *P* < 0.001). There was no significant difference in the other data between the three groups.

**Conclusion:**

Compared with the RS group, the WS group had an increased risk of local recurrence. However, the WS group had a similar DFS and OS compared with the RS group and the local excision group. Hence, we speculated that the WS group would have similar results as the surgery group for patients with cCR status.

## Background

Colorectal cancer is a serious threat to human health. Mid-low rectal cancer accounts for 70% of colorectal cancer [1, 2]. Radical surgery (RS) remains the main treatment for mid-low rectal cancer [3, 4]. However, 10–20% of patients still have local recurrence after radical surgery [5, 6]. Neoadjuvant chemoradiotherapy has reduced the local recurrence rate of patients and can preserve the anus. Approximately 20% of rectal cancer patients have a good response to neoadjuvant chemoradiotherapy (NCRT). If NCRT (50.4 Gy/25 and capecitabine) is performed, surgery should be extended approximately 6–12 weeks. The tumor can shrink to a minimum size and even disappear completely after neoadjuvant chemoradiotherapy [7]. Approximately 10–20% of patients can achieve a clinical complete response (cCR) [8]. The stringent definition of cCR was proposed by Mass in 2012, and it contained five diagnostic criteria [9]. The diagnostic criteria of cCR were no residual tumor and white scar in endoscopy, negative biopsies from the white scar, no palpable tumor with digital rectal exam (DRE), no suspicious lymph nodes in MRI, and substantial downsizing with no residual tumor or residual fibrosis in MRI [10]. In our opinion, the most critical points in adopting the wait and see strategy (WS) strategy are no residual tumor in endoscopy and no suspicious lymph nodes or residual tumor in MRI.

In 2004, Habr-Gama published the results of rectal cancer patients with cCR status after neoadjuvant chemoradiotherapy. Habr-Gama first proposed the WS strategy of treating rectal cancer patients with cCR status and pointed out that the WS strategy could achieve similar clinical effects as surgery [11]. The 5-year DFS and OS rates of cCR status were 92% and 100%, respectively. The 5-year disease-free and 5-year overall survival rates of the radical surgery group were 83% and 88%, respectively. No significant differences were found between the two groups. Nowadays, there are three routine treatments for patients with cCR status: they are WS strategy, radical surgery, and local resection. WS strategy has no invasive operations to the tumor, no surgical trauma, and no change in intestinal function, which ensures the good quality of life of patients. Radical surgery mainly follow the total mesorectal excision (TME) principle, where it needs to remove 15 cm bowel from the upper edge of the tumor and 3–5 cm from the lower edge, with the corresponding lymph nodes. After radical surgery, there may be some changes in bowel function with increased stool frequency and decreased sexual function. And some patients may have loose anus. It could bring physical and psychological trauma to the patients. Local excision only cut off the tumor and the surrounding normal tissues to ensure negative margins, and it could ensure the continuity of the intestine and would not have a significant impact on bowel function and quality of life. The details of specific resection range are in additional file 8.

There were several meta-analyses related to this topic, such as Li’s research, Dossa’s research, and some other papers published in a Chinese magazine [12–14]. The disease-free survival (HR 0.56, 95% CI 0.20–1.60) and overall survival (HR 3.91, 95% CI 0.57–26.72) of Dossa’s research are similar to those of Li. Our study included more studies and more patients to explore the efficacy and safety of the wait and see strategy versus those of surgery for rectal cancer with cCR response after neoadjuvant chemoradiotherapy. We also performed a meta-analysis to compare the efficacy and safety of the WS strategy versus those of radical surgery and those of WS strategy versus those of local resection (LE).

## Methods

### Literature search

We carried out this meta-analysis by using the PRISMA guidelines. The details of PICOS were as follows: Population: rectal cancer patients with a cCR response after neoadjuvant chemoradiotherapy; Intervention: wait and see strategy; Comparator: radical surgery or local excision; Outcomes: long-term outcomes including local recurrence, distant metastasis, cancer-related death, disease-free survival (DFS), and overall survival (OS) which were analyzed and compared (additional file 3). Continuous data were evaluated by the standardized mean differences (SMD) with 95% confidence intervals (95% CIs), and dichotomous outcomes were evaluated by relative risks (ORs or RRs) with 95% CIs.

We searched the Cochrane Library, PubMed, CNKI (China National Knowledge Infrastructure), and Wanfang databases (up to March 2020). Articles about wait and see versus radical surgery or local excision after neoadjuvant chemoradiotherapy for rectal cancer were collected. To avoid missing useful articles, we expanded the scope of search terms and find articles for the purpose by manual screening. The search terms were “wait and see” or “nonoperative management” and “neoadjuvant chemoradiotherapy” and “rectal cancer.” The details are shown in additional file 1.

### Inclusion and exclusion criteria

There were 4 inclusion criteria: (1) pathological and long-term outcomes were compared between wait and see and radical surgery or local excision for rectal cancer with cCR response after neoadjuvant chemoradiotherapy; (2) surgery included radical surgery and local excision; (3) cCR response after neoadjuvant chemoradiotherapy (criteria for ccR were mentioned above) [15]; and (4) RCT (randomized Controlled Trial), RCNTs (retrospective comparative non-randomized studies), PCNTs (prospective comparative non-randomized studies), cohort studies, or case-control studies. The details of diagnostic criteria of all studies are shown in Table 1. The details of diagnostic criteria of each method in each study are shown in additional file 2.

**Table 1:** Characteristics of the included articles

Study	Year	Country	Case			Age			Quality control			Diagnostic criteria	Study design	Neoadjuvant therapy	Evaluation	Follow-up time (month)			Radical surgery type	NOS score
			WS	RS	LE	WS	RS	LE	Selection	Comparability	Outcome				Time (week)	WS	RS	LE	APR or LAR	
Ayloor [16]	2013	India	23	10	–	50	55	–	3	2	1	➀➁➂+ CT, ultrasound	RNCT	Long-range radiotherapy	4–6	72	72	–	APR or LAR	6
Dalton [17]	2012	UK	6	6	–	68	69	–	3	2	2	➀➁➃➄	PNCT	Cape/45–50.4Gy	6–8	25.3	39.3	–	TME	7
Habr [11]	2004	Brazil	71	22	–	58.1	53.6	–	2	3	1	➀➁➂+CT	PNCT	5-FU+LV/45–50.4Gy	6–8	57.3	48	–	TME	6
Lai [18]	2016	Taiwan	18	26	–	67.5	63.7	–	2	2	2	➀➃➄	RNCT	5-FU/45–50.4Gy	8–12	49	42	–	APR or LAR or LAR+ loop stoma	6
Lee [19]	2015	Korea	8	28	16	64	70	70	3	2	1	➂➃➄	PNCT	50.4Gy	6–10	41	41	41	TME	6
Li [20]	2015	China	30	92	–	62	56	–	3	1	2	➀➁➂➃➄	PNCT	Cape/50;25Gy	8–10	58	58	–	APR or LAR	6
Mass [21]	2011	Netherlands	21	20	–	65	66	–	3	2	2	➀➁➂➃➄	PNCT	Cape/45Gy	6–8	25	35	–	TME	7
Renehan [22]	2016	UK	129	109	–	66.9	65	–	2	3	2	➀➁➂➃➄	PNCT	5-FU/45Gy	8	33	33	–	TME	7
Smith [23]	2012	USA	32	57	–	70	60	–	3	3	1	➀➁➂➃➄	PNCT	5-FU+ Cape/50.4Gy	4–10	28	43	–	–	7
Yeom [24]	2019	Korea	15	129	25	74	64.8	73	2	3	2	➀➁➂➃➄	RNCT	Cape /50.4Gy or Capeox/50.4Gy or 5-FU /50.4Gy	8	60	60	60	TME	7
Wang [25]	2020	China	59	179	–	58	57	–	2	2	2	➀➁➂➃➄	RNCT	5-FU+ Cape/50.4Gy	6–12	60	60	–	TME	6

Notes: *PNCT* prospective non-randomized controlled trial, *RNCT* retrospective non-randomized controlled trial, *5-FU* 5-fluorouracil, *Cape* capecitabine, *RS* radical surgery, *TME* total mesorectal excision, *APR* abdomi-l-perineal resection, *LAR* low anterior resection

Diagnostic criteria of cCR: ➀ no residual tumor and white scar in endoscopy, ➁ negative biopsies from the white scar, ➂ no palpable tumor with digital rectal exam (DRE), ➃ no suspicious lymph nodes in MRI, and ➄ no residual tumor or residual fibrosis in MRI

There were 3 exclusion criteria: (1) studies with no valuable outcome; (2) patients were not well grouped or groups were confusing and not suitable for the purpose of the article; and (3) bad clinical response of rectal cancer after neoadjuvant chemoradiotherapy. Bad clinical response included larger residual tumor in endoscopy and DRE, positive biopsies, and larger tumor and lymph nodes in MRI. The details of inclusion and exclusion criteria are in additional file 6.

### Data extraction and quality control

Using the Newcastle-Ottawa Scale (NOS) guidelines, two reviewers searched the literatures (GHZ and DMY) independently [26]. We collected the useful data, as shown in Tables 1, 2, 3, and 4. Table 1 contains the baseline data of related studies. Table 2 contains the initial tumor stages of the included patients. Table 3 contains the tumor staging after including neoadjuvant therapy. Table 4 contains the primary and secondary objectives. The primary objectives include local recurrence (LE), distant metastasis (DM), and cancer-related death (CRD). The secondary objectives include disease-free survival (DFS) and overall survival (OS). More basic information of patients and the details of neoadjuvant treatment plans are in additional file 4 and additional file 5. A third reviewer had the final decision power to resolve the disagreements of the study. We tried to contact the authors with missing data, but did not get any relevant data.

**Table 2:** T stage, N stage and clinical stage of the included articles

Study	T stage (*n*; %)						N stage (*n*; %)					
	T1–T2			T3–T4			N0			N1–N2		
	WS	RS	LE	WS	RS	LE	WS	RS	LE	WS	RS	LE
Ayloor [16]	9	4	–	14	6	–	–	–	–	–	–	–
Dalton [17]	1	–	–	5	6	–	1	–	–	5	6	–
Habr [11]	14	1	–	57	21	–	55	16	–	16	6	–
Lai [18]	–	–	–	–	–	–	–	–	–	–	–	–
Lee [19]	5	6	7	3	22	9	5	13	14	3	15	2
Li [20]	8	24	–	22	68	–	14	39	–	16	53	–
Mass [21]	6	1	–	15	19	–	6	3	–	15	17	–
Renehan [22]	31	24	–	98	85	–	45	47	–	84	181	–
Smith [23]	10	11	–	22	39	–	14	20	–	18	31	–
Yeom [24]	3	8	2	12	121	23	5	75	19	10	54	6
Wang [25]	6	8	–	53	171	–	14	47	–	45	132	–
Total	93 (23.6)	87 (13.3)	9 (21.9)	301 (76.3)	558 (85.5)	32 (78.1)	159 (42.8)	260 (34.4)	33 (80.4)	212 (57.2)	495 (65.5)	8 (19.6)
Clinical stage (*n*; %)												
I			II			III				IV		
WS	RS	LE	WS	RS	LE	WS	RS	LE		WS	RS	LE
–	–	–	–	–	–	–	–	–		–	–	–
–	–	–	–	–	–	–	–	–		–	–	–
–	–	–	–	–	–	–	–	–		–	–	–
–	–	–	11	8		7	18	–		–	–	–
–	–	–	–	–	–	–	–	–		–	–	–
–	–	-	–	–	–	–	–	–		–	–	–
–	–	–	–	–	–	–	–	–		–	–	–
–	–	–	–	–	–	–	–	––		–	–	–
8	2	–	6	18	–	18	31	–		–	–	–
–	–	–	–	–	–	–	–	–		–	–	–
–	–	–	–	–	–	–	–	–		–	–	–
8 (16)	2 (2.5)	–	17 (34)	26 (33.7)	–	25 (50)	49 (63.8)	–		–	–	–

**Table 3:** Pathologic staging and subtypes of included articles after radiotherapy

Study	Pathologic T stage (*n*; %)										Pathologic N stage (*n*; %)			
	ypT0		ypT1		ypT2		ypT3		ypT4		ypN0		ypN1–2	
	RS	LE	RS	LE	RS	LE	RS	LE	RS	LE	RS	LE	RS	LE
Ayloor [16]	6	–	–	–	3	–	1	–	–	–	6	–	5	–
Dalton [17]	–	–	–	–	–	–	–	–	–	–	–	–	–	–
Habr [11]	–	–	–	–	–	–	–	–	–	–	–	–	–	–
Lai [18]	–	–	–	–	–	–	–	–	–	–	–	–	–	–
Lee [19]	13	6	2	6	9	4	4	0	0	0	24	–	4	–
Li [20]	–		–		–	–		–	–	–	–	–	–	–
Mass [21]	–		–		–	–		–	–	–	–	–	–	–
Renehan [22]	–		–		–	–		–	–	–	–	–	–	–
Smith [23]	–		–		–	–		–	–	–	–	–	–	–
Yeom [24]	46	12	47	11	–	–	36	2	–	–	108	–	21	–
Wang [25]	–		–		–	–		–	–	–	–	–	–	–
Total	65 (38.9)	18 (43.9)	49 (29.3)	17 (41.4)	12 (7.1)	4 (9.7)	41 (24.7)	2 (5)	0	0	138 (82.1)		30 (17.9)	–
Pathologic subtypes														
Well differentiated		Moderate differentiated				Poorly differentiated						Unknown		
WS	RS	WS		RS		WS			RS			WS	RS	
–	–	–		–		–			–			–	–	
–	–	–		–		–			–			–	–	
–	–	–		–		–			–			–	–	
–	–	–		–		–			–			–	–	
–	–	–		–		–			–			–	–	
–	–	–		–		–			–			–	–	
–	–	–		–		–			–			–	–	
5	15	86		104		2			14			36	95	
–	–	–		–		–			–			–	–	
–	–	–		–		–			–			–	–	
2	29	45		126		2			0			10	24

**Table 4:** Characteristics of the included articles

Study	LR (*n*/%)			DM (*n*/%)			CRD (*n*/%)				
	WS	RS	LE	WS	RS	LE	WS	RS	LE		
Ayloor [16]	7	0	–	3	2	–	–	–	–		
Dalton [17]	–	–	–	–	–	–	–	–	–		
Habr [11]	2	0	–	3	3	–	0	2	–		
Lai [18]	2	0	–	0	1	–	–	–	–		
Lee [19]	2	1	6	0	3	2	–	–	–		
Li [20]	2	2	–	1	5	–	0	4	–		
Mass [21]	1	0	–	0	1	–	–	–	–		
Renehan [22]	–	–	–	–	–	–	–	–	–		
Smith [23]	6	0	–	3	3	–	1	0	–		
Yeom [24]	6	15	5	4	5	4	–	–	–		
Wang [25]	7	1	–	6	17	–	–	–	–		
Total	35 (12.6)	19 (3.3)	11 (26.8)	20 (7.2)	40 (7.1)	6 (14.6)	1 (0.7)	6 (3.5)	–		
2-year OS (*n*/%)		2-year DFS (*n*/%)			5-year OS (*n*/%)		5-year DFS (*n*/%)				
WS	RS	LE	WS	RS	LE	WS	RS	LE	WS	RS	LE
–	–	–	–	–	–	–	–	–	–	–	–
6	6	–	6	6	–	–	–	–	–	–	–
71	20	–	70	19	–	71	20	–	68	19	–
18	26	–	–	–	–	18	24	–	–	–	–
–	–	–	6	25	10	–	–	–	–	–	–
30	92	–	29	91	–	30	88	–	27	85	–
21	19	–	19	19	–	–	–	–	–	–	–
107	100	–	100	89	–	–	–	–	–	–	–
31	57	–	28	56	–	–	–	–	–	–	–
–	–	–	–	–	–	3	128	–	3	110	–
–	–	–	–	–	–	53	175	–	–	–	–
284 (92.5)	320 (96.3)	–	258 (86.8)	305 (91.3)	10 (62.5)	175 (90.6)	435 (97.0)	–	98 (84.4)	214 (88.0)	–

Notes: *LR* local recurrence, *DM* distant metastasis, *CRD* cancer-related death, *DFS* disease-free survival, *OS* overall survival

### Statistical analysis

We used RevMan 5.0 to carry out the meta-analysis. Continuous data were evaluated by the standardized mean differences (SMD) with 95% confidence intervals (95% CIs), and dichotomous data were evaluated by relative risks (ORs or RRs) with 95% CIs. Heterogeneity and publication bias were estimated by *I*^2^ statistic and funnel plots separately. When huge heterogeneity existed (*I*^2^≧50%), we used random-effects models to analyze the data. We used fixed-effects model to analyze the data with little heterogeneity (*I*^2^ < 50%).

## Results

### Study selection

**Fig. 1:**
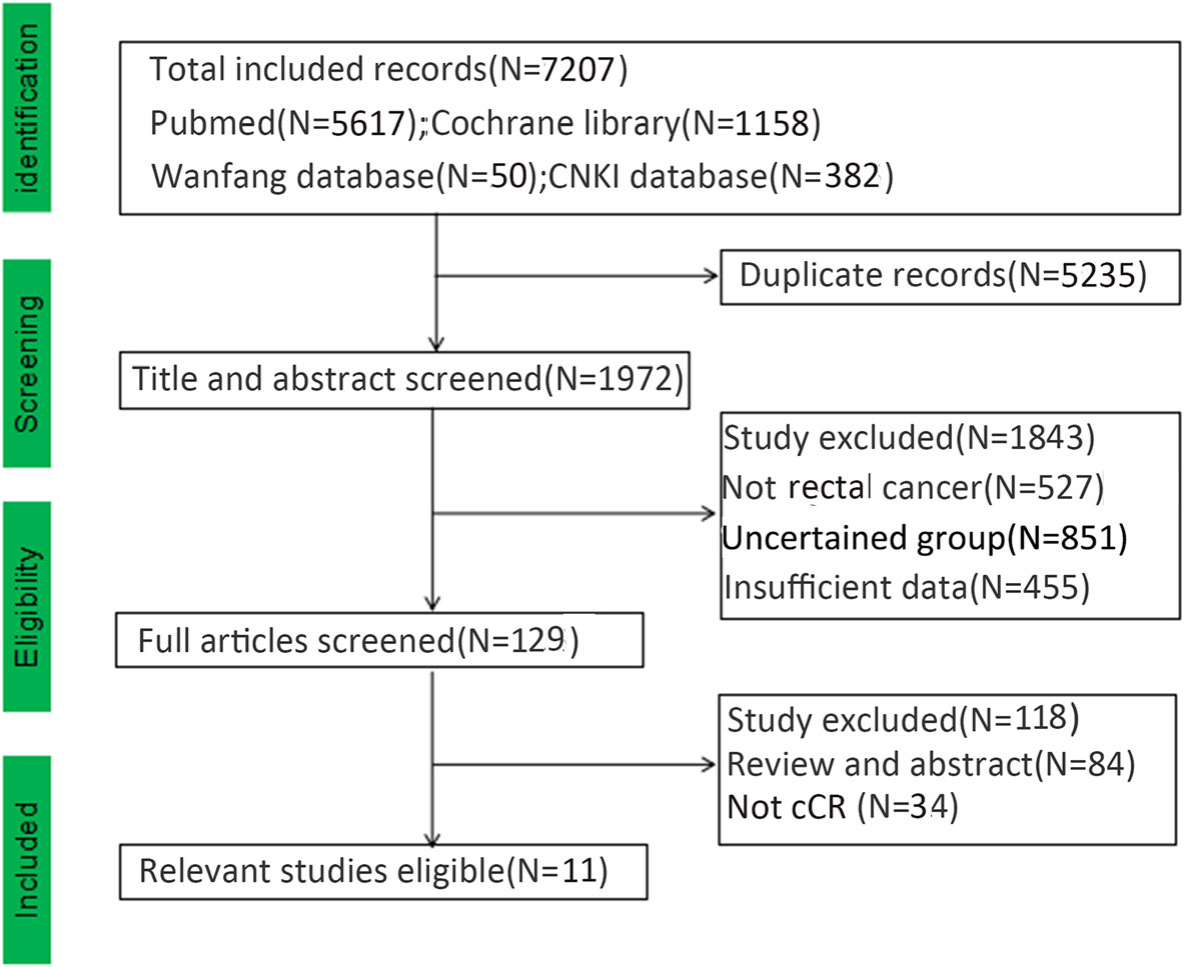
Flowchart of the included studies

Duplicated records were deleted. We deleted 1843 studies after reading the titles and abstracts carefully. Deleted studies were due to not of rectal cancer (*n* = 527), no control group (*n* = 851), and insufficient data (*n* = 455). Regarding study information, after we read the remaining studies carefully, 11 English studies with 1131 patients were included [11, 16–25]. There were 412 patients in the WS group, 678 patients in the RS group, and 41 patients in the LE group. There were 6 Eastern studies and 5 Western studies in the meta-analysis. There were 10 English studies, and 1 was a Chinese study. The Western research included European, American, and Latin American research, while the Eastern research mainly included Asian research. Yeom and Lai reported local resection for rectal cancer with cCR response after neoadjuvant chemoradiotherapy. Some baseline data from the articles were inconsistent, which could affect the results. We deleted several articles and hoped to reduce the bias caused by inconsistent baseline data. Patient information: the clinical stages of included patients were stages I to III. Approximately 81.9% of patients were at the beginning T3–4 and 67.8% N1–2 (Table 2). A total of 19.4% of patients were ypT3–T4 and 11.8% of patients were ypN1–2, and we divided the surgery group into RS (radical surgery) and LE (local excision). The clinical staging of included articles after neoadjuvant chemoradiotherapy is shown in Table 3. The long-term outcomes are shown in Table 4. The long-term outcomes included local recurrence, distant metastasis, cancer-related death, disease-free survival, and overall survival (Fig. 1).

### Quality assessment

We evaluated the quality of the included studies using the NOS assessment scale (Newcastle-Ottawa Quality Assessment Scale). The score indicated three levels of quality: low (1–3), moderate (4–6), and high (7–9). We included 11 studies in our study (4 RCNT and 7 prospective non-randomized controlled trial (PNCT)) with moderate to high quality. No relevant RCTs were found during the database search. The details are shown in Table 1.

## Primary objectives

### WS group versus RS group

#### Local recurrence, distant metastasis, and cancer-related death

**Fig. 2:**
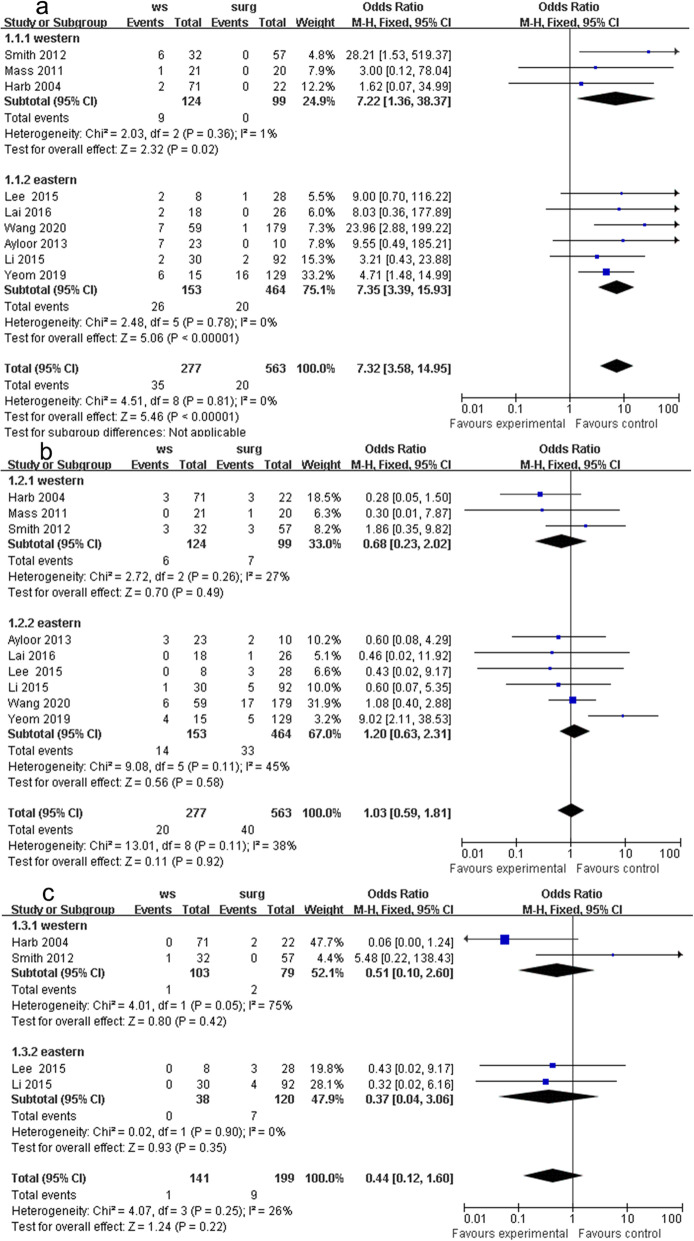
Outcomes of WS group versus Radical surgery group. **a** local recurrence, **b** distant metastasis, and **c** cancer-related death

The WS group had a higher recurrence rate than the RS group. Other primary objectives were similar in the two groups. The details were as follows: 9 studies reported clinical data on local recurrence, and the WS group had a higher recurrence rate than the RS group in the fixed-effects model (OR 7.32, 95% CI 3.58 to 14.95, *P* < 0.001, chi^2^ = 4.51, *P* = 0.81, *I*^2^ = 0%, Fig. 2a). Western studies (OR 7.22, 95% CI 1.36 to 38.37, *P* = 0.02, chi^2^ = 2.03, *P* = 0.36, *I*^2^ = 1%, Fig. 2a), and Eastern studies (OR 5.57, 95% CI 2.36 to 13.15, *P* < 0.001, chi^2^ = 0.69, *P* = 0.95, *I*^2^ = 0%, Fig. 2a) had the same results in local recurrence. The WS group had a similar distant metastasis rate as the radical surgery group in the fixed-effects model with high heterogeneity (OR 1.03, 95% CI 0.59 to 1.81, *P* = 0.92, chi^2^ = 13.01, *P* = 0.11, *I*^2^ = 38%, Fig. 2b). The occurrence of cancer-related death (OR 0.44, 95% CI 0.12 to 1.60, *P* = 0.22, chi^2^ = 4.07, *P* = 0.25, *I*^2^ = 26%, Fig. 2c) was similar between the two groups in the fixed-effects model with little heterogeneity.

### WS group versus LE group

#### Distant metastasis and local recurrence

**Fig. 3:**
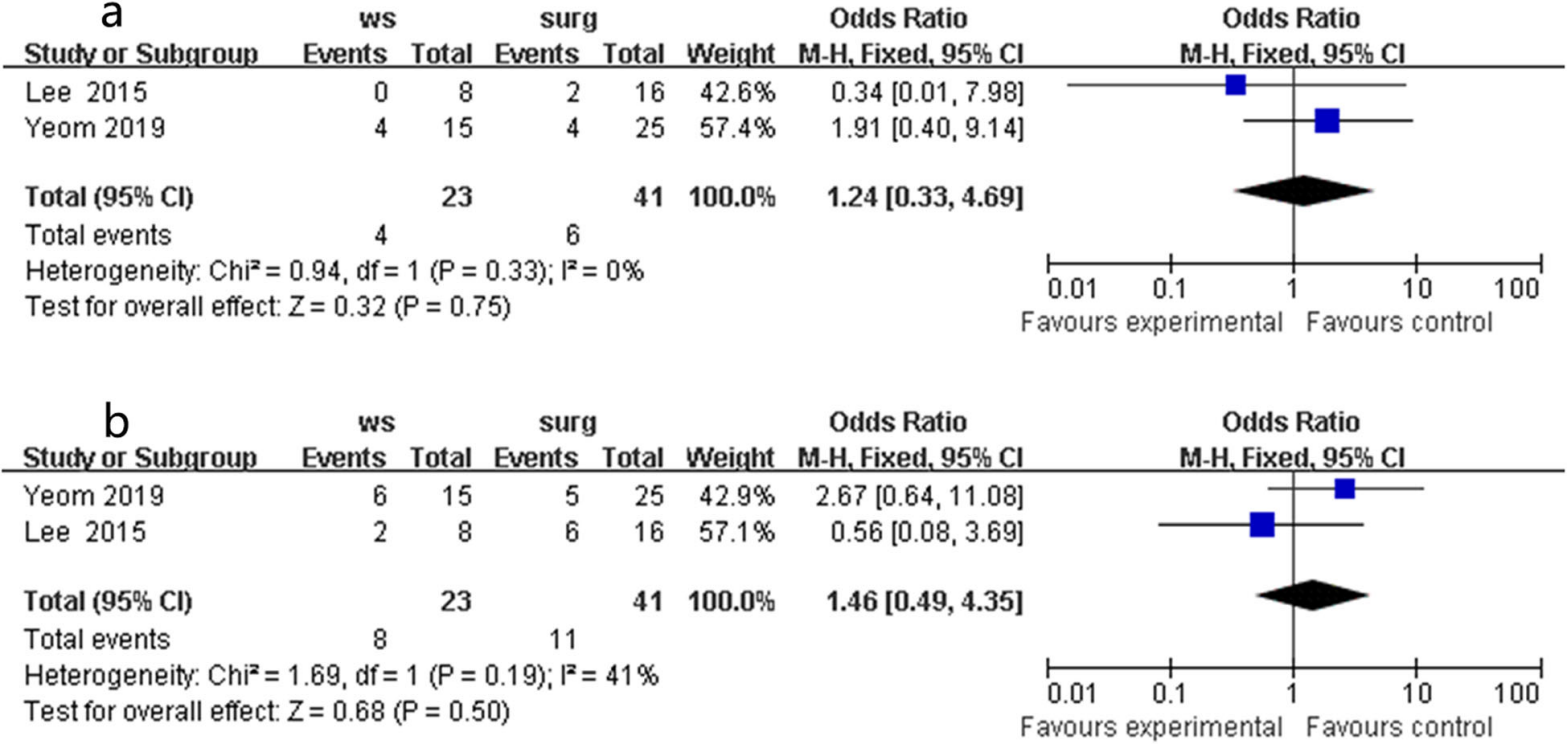
Outcomes of WS group versus local excision group. **a** distant metastasis and **b** local recurrence

The primary objectives were similar in the two groups. Similar distant metastasis rates were found in the two groups in the fixed-effects model with little heterogeneity (OR 1.24, 95% CI 0.33 to 4.69, *P* = 0.75, chi^2^ = 0.94, *P* = 0.33, *I*^2^ = 0%, Fig. 3a). Two studies reported clinical data on local recurrence, which was similar in the two groups in the fixed-effects model (OR 1.46, 95% CI 0.49 to 4.35, *P* = 0.50, chi^2^ = 1.69, *P* = 0.19, *I*^2^ = 41%, Fig. 3b).

## Secondary objectives

### WS group versus RS group

#### 2-Year DFS, 2-year OS, 5-year DFS, and 5-year OS

**Fig. 4:**
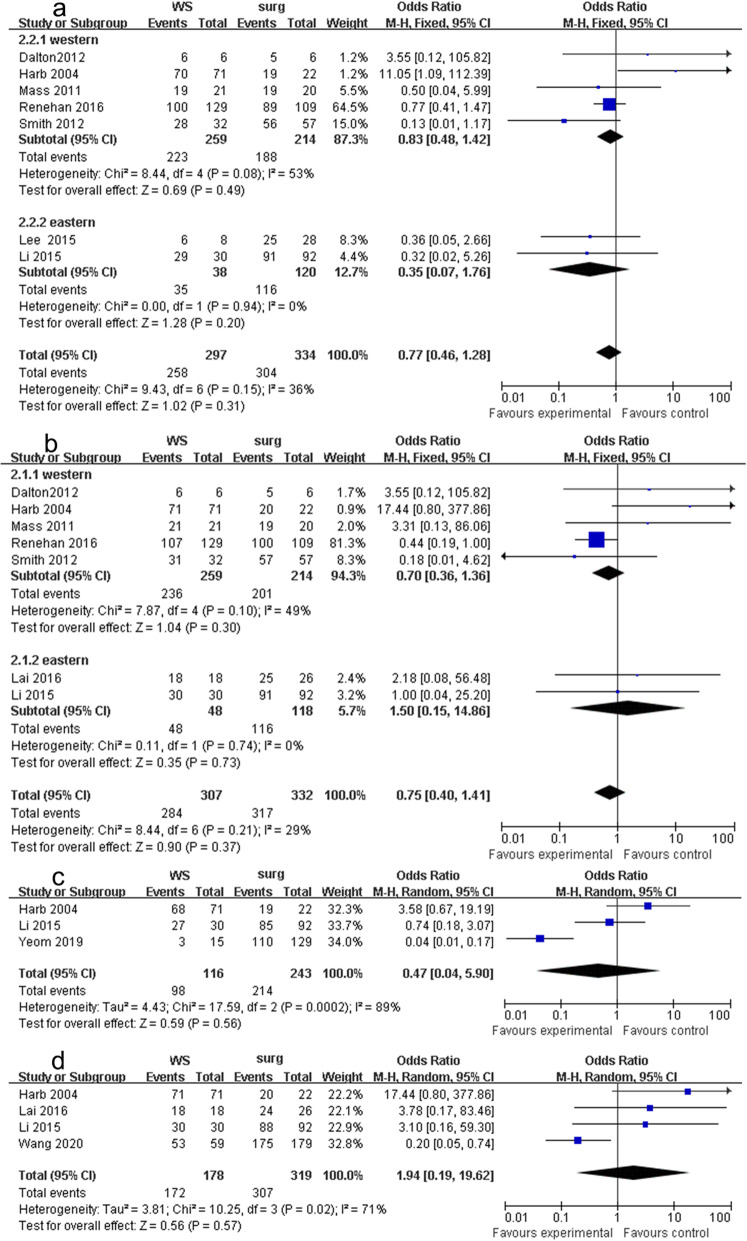
Outcomes of WS group versus radical surgery group. **a** 2-year DFS, **b** 2-year OS, **c** 5-year DFS, and **d** 5-year OS

The secondary objectives were similar in two groups. The details are as follows: seven studies with WS group had similar 2-year DFS as the RS group in the fixed-effects model with little heterogeneity (OR 0.77, 95% CI 0.46 to 1.28, *P* = 0.3, chi^2^ = 9.44, *P* = 0.15, *I*^2^ = 36%, Fig. 4a). Seven studies that reported OS with WS groups had similar 2-year OS as the surgery group in the fixed-effects model with little heterogeneity (OR 0.75, 95% CI 0.40 to 1.41, *P* = 0.90, chi^2^ = 8.44, *P* = 0.21, *I*^2^ = 29%, Fig. 4b). The 5-year DFS (OR 0.47, 95% CI 0.04 to 5.90, *P* = 0.56, chi^2^ = 17.59, *P* < 0.001, *I*^2^ = 89%, Fig. 4c) and 5-year OS (OR 1.94, 95% CI 0.19 to 19.62, *P* = 0.56, chi^2^ = 10.25, *P* = 0.02, *I*^2^ = 71%, Fig. 4d) were similar in both groups in the random-effects model.

### Publication bias

To determine whether the article had publication bias, we used RevMan 5.0 software to test the index of distant metastasis rate of the included literature and obtained a funnel plot. The points were evenly distributed in the funnel plot, indicating no publication bias in the meta-analysis.

## Discussion

Radical surgery of mid-low rectal cancer can bring great trauma to patients, and some patients require removal of their anus [27], which could result in a serious impact on patients’ physical and mental health [28]. In 2004, Habr-Gama first reported the wait and see (non-surgical) treatment strategy for low rectal cancer patients who obtained cCR after chemoradiotherapy. A total of 71 patients who achieved a cCR status were only closely followed (wait and see) for an average of 57.3 months. Two patients had recurrence in the intestinal lumen, and 3 patients had distant metastasis. The 5-year DFS and OS rates were 92%, and 100% respectively. The other 194 patients without clinical complete response underwent radical surgery. A total of 22 patients had pathologically confirmed pCR (pathologically complete response). The 5-year disease-free survival rate and 5-year overall survival rates were 83% and 88%, respectively. No significant differences were found between the two groups. After expanding the sample size, Habr-Gama got the same conclusion [29]. Subsequently, many studies about cCR have emerged, and they also confirmed the treatment effect of the wait and see strategy [30, 31]. Professor Lu found that 50% of cT1–2 tumors could disappear completely, and postoperative pathology was ypT0 [32]. In addition, as the tumor shrank, local excision surgery and the wait and see strategy were able to ensure organ preservation. Therefore, we aimed to include cCR patients and intended to compare the advantages and disadvantages of the three treatment methods.

### Local recurrence, distant metastasis, and cancer-related death

In this meta-analysis, we found that the WS group had a higher local recurrence rate than the RS group. This result was similar to professor Li’s research. Approximately 81.9% of patients were at the beginning T3–4 and 67.8% N1–2 (Table 2). A total of 19.4% of patients were ypT3–T4 and 11.8% of patients were ypN1–2. Patients in whom T stage and N stage were not obviously reduced were prone to relapse after neoadjuvant therapy. Patients could relapse in the WS group easily. However, in the RS surgery group, the recurrence rate was reduced to below 10%. Because few patients had incomplete information in LE group, no significant difference in local recurrence rate was seen in the WS and LE group. We speculated that some patients achieved cCR status in the WS group, but the tumor was not completely cured, so the recurrence rate would increase. But in the surgery group, the tumor has been removed and the recurrence rate can be appropriately reduced. Approximately 7.2% of patients had distant metastasis in the WS group and 7.1% of patients had distant metastasis in the RS group. Fewer studies involved the CRD indicator. There were no significant differences of distant metastasis rate and CRD in three groups. Dossa and Li also arrived at the same result. The wait and see strategy had some advantages, such as reducing surgical trauma, improving the quality of life, and no raising distant metastasis rate.

### DFS and OS

The 2-year DFS rate was 86.8% and 91.3% in the WS and RS groups, respectively. The 2-year OS rates were 92.5% and 96.3% in the WS and RS groups, respectively. The WS group had similar 2-year DFS and OS and 5-year DFS and OS as the RS group. Patients with cCR status were sensitive to neoadjuvant therapy and had good biological behavior, so patients’ survival was good in all groups. Due to the huge difference in the baseline data, we deleted Lin’s study [33]. We obtained similar results as Li’s research and Dossa’s research. Lin’s study caused big bias and led to the false result. Arauio and Simth’s research included patients with tumor stage IV, so we abandon them [34, 35]. Currently, doctors use high-sensitivity MRI and colonoscopy to find small tumor lesion and deal with them in time [36, 37]. The included articles had salvage therapy for patients with local recurrence, which included chemotherapy and surgical treatment. The patients of the WS group, who had not experienced large surgical trauma, had better immunity than surgical group, and they could endure the subsequent treatments.

### The specific plan and efficacy of NRCT

The details of the neoadjuvant treatment are shown in additional file 5. The NCRT of Li’s research (50 Gy/25 f/2 Gy, capecitabine, 825 mg/m2 bid, concurrently) had the best clinical effect. This research had the biggest PCR rate and the best DFS/OS rate with long-term radiotherapy and capecitabine. Professor Harb also conducted long-term radiotherapy + 5-fluoracil for neoadjuvant therapy, with a PCR rate of approximately 8.3%. Due to the improvement in the level of radiotherapy and the use of capecitabine sensitizers, the PCR rate was about 20%. At present, the standard neoadjuvant treatment is radiotherapy (45 to 50.4 Gy/25 to 28 f) and capecitabine at 825 mg/m^2^, twice per day (total dose of 1 650 mg/m^2^/d). Surgery should be approximately about 6–8 weeks and 12 weeks at the maximum. In this way, the tumor can shrink to a minimum and the edema of the irradiation field completely disappeared, improving the PCR and anus preservation rate [38]. The included literature had a long time span, and the neoadjuvant treatment plan was not uniform, which could affect the final result. With the further standardization of neoadjuvant treatment, we hoped more and more studies with the uniform adjuvant treatment plans would appear.

### Previous meta-analysis and our update

Although Professor Dossa included 23 studies in his research, only 15 studies had control groups and 5 studies had specific data of comparison between two groups. He proposed that there were no significant differences in non-regrowth recurrence, cancer-specific mortality, overall survival, and disease-free survival. There were 9 studies in Professor Li’s research, and he reported that two patient groups were similar in distant metastasis rates, disease-free, and overall survival, but the non-surgical group had a higher risk of 1-, 2-, 3-, and 5-year local recurrence. Our report also showed a higher local recurrence rate in the WS group than the RS group, which affirmed the effect of wait and see strategy for patients with cCR status. Subgroup analysis of Eastern and Western studies were also performed, and we hoped that more studies about cCR-related research of the WS group versus radical surgery or local excision would appear [39].

Arauio and Smith had patients with Stage IV; thus, we removed those two studies. More cases with T3–4 and N1–2 stage are more needed to perform neoadjuvant treatment than T1–2 and N0 stage. We found that some patients with T3–4 and N1–2 stage could obtain cCR after neoadjuvant treatment with better prognosis than adjuvant treatment. Sometimes, T1–2 and N0 stage might be treated directly with surgery and obtained a considerable effect. However, the relevant prognosis of patients with T and N stage were not specified in the article, and we could not use the meta-analysis to define the specific meaning of T and N stage for cCR. Most patients had achieved T and N stage reduction after neoadjuvant therapy, but there was too little available information to form a conclusion about a specific treatment method for these patients.

## Limitations

This study might have several limitations. First, 11 studies (4 RCNT and 7 PNCT) with a total of 1131 patients could not represent the highest level of evidence due to the non-included RCTs. Second, incomplete clinical data and some unbalanced baseline characteristics could affect the results. Third, differences between Eastern and Western population groups could cause potential selection bias. Finally, more cCR patients need to be included. Furthermore, more RCTs for the wait and see strategy versus surgery for cCR are necessary.

## Conclusion

In summary, this study compared the reliability and safety of the wait and see strategy to radical surgery and local excision for rectal cancer with cCR response after neoadjuvant chemoradiotherapy. The WS group had higher local recurrence rate than the RS group. There was no significant difference in other data. With neoadjuvant chemoradiotherapy development and appropriate salvage therapy for local recurrence, the wait and see strategy could minimize surgical trauma and preserve the anus and had the advantages for cCR patient. Therefore, we propose that the wait and see strategy could be a feasible model for cCR patient.

## Supplementary information

**Additional file 1:.**

**Additional file 2:.** The details of diagnostic criteria of each method in each study.

**Additional file 3:.** The details of PICOS

**Additional file 4:.**

**Additional file 5:.** The details of neoadjuvant treatment of studies

**Additional file 6:.** The details of Inclusion and Exclusion Criteria

**Additional file 7:.**

**Additional file 8:.**

## Data Availability

All data generated or analyzed during this study are included in this published article.

## Abbreviations

PNCT: Prospective non-randomized controlled trial RNCT Retrospective non-randomized controlled trial 5-FU 5-Fluorouracil Cape Capecitabine RS Radical surgery TME Total mesorectal excision APR Abdominal-perineal resection LAR Low anterior resection CI Confidence interval OR Odds ratios OS Overall survival SMD Standardized mean difference
