# Supplementary material for: Efficacy and safety of wait and see strategy versus radical surgery and local excision for rectal cancer with cCR response after neoadjuvant chemoradiotherapy: a meta-analysis
Source: World J Surg Oncol. 2020 Aug 31;18:232. doi: 10.1186/s12957-020-02003-6 (PMC7457353; doi:10.1186/s12957-020-02003-6)
Supplement: Supplementary file 1 — Additional file 1:. [file 12957_2020_2003_MOESM1_ESM.doc]

The search term is as follows:

(nonoperative[All Fields] AND ("organization and administration"[MeSH Terms] OR ("organization"[All Fields] AND "administration"[All Fields]) OR "organization and administration"[All Fields] OR "management"[All Fields] OR "disease management"[MeSH Terms] OR ("disease"[All Fields] AND "management"[All Fields]) OR "disease management"[All Fields])) OR (wait[All Fields] AND ("Tydskr Stud Ekon Ekon"[Journal] OR "see"[All Fields]) AND ("rectal neoplasms"[MeSH Terms] OR ("rectal"[All Fields] AND "neoplasms"[All Fields]) OR "rectal neoplasms"[All Fields] OR ("rectal"[All Fields] AND "cancer"[All Fields]) OR "rectal cancer"[All Fields]) AND ("neoadjuvant therapy"[MeSH Terms] OR ("neoadjuvant"[All Fields] AND "therapy"[All Fields]) OR "neoadjuvant therapy"[All Fields] OR "neoadjuvant"[All Fields]) AND ("chemoradiotherapy"[MeSH Terms] OR "chemoradiotherapy"[All Fields]))
