# Supplementary material for: Efficacy and safety of wait and see strategy versus radical surgery and local excision for rectal cancer with cCR response after neoadjuvant chemoradiotherapy: a meta-analysis
Source: World J Surg Oncol. 2020 Aug 31;18:232. doi: 10.1186/s12957-020-02003-6 (PMC7457353; doi:10.1186/s12957-020-02003-6)
Supplement: Supplementary file 2 — Additional file 2:. The details of diagnostic criteria of each method in each study. [file 12957_2020_2003_MOESM2_ESM.doc]

The details of diagnostic criteria of each method in each study.

Ayloor 2013

All the patients were clinically reassessed 4-6 weeks after completion of NCRT. The response to neoadjuvant treatment was assessed by DRE and a CT scan. All patients with a suspected cCR also underwent a sigmoidoscopy with or withour biopsy. For the purpose of this study, patients were considered to have cCR if there was no documented residual tumor in the rectum on DRE and sigmoidoscopy. Patients in whom the response was documented as a minimal induration in the rectal wall or mucosal irregularity without mucosal ulceration were considered as having cCR and included in this study.

Dalton2012

A repeat MRI scan is undertaken initially at 6 weeks (more recently changed to 8 weeks) post-CRT with further multidisciplinary team discussion and surgery planned as appropriate if tumour is considered to be present. TME is undertaken for middle and lower rectal tumours. If on MRI there has been significant tumour regression with little evidence of residual tumour, then further investigations are conducted, looking for evidence of residual disease, following extensive discussion with the patient. An examination under anaesthesia (EUA) is undertaken, with biopsies of any residual scar tissue. Any residual mucosal ulcer is considered to be residual tumour even if biopsies are benign. If there is no evidence of tumour clinically and on biopsy, an FDG-PET scan is carried out. Patients with evidence of residual disease proceed to surgery, with disease-free patients considered as having complete clinical response (CCR) at this stage.

Patients with CCR are followed up with a repeat EUA at 3 months and at 1 year, with initially 6-monthly and then yearly PET/CT and MRI. Carano-embryonic antigen levels are also monitored. Patients are fully informed that tumour may be detected during follow up and if so will require surgery.

Habr 2004

After 8 weeks from completion of CRT, patients were reevaluated by an experienced colorectal surgeon to assess tumor response using the same pretreatment clinical, endoscopic, and radiologic parameters. During proctoscopy, biopsies were obtained for pathologic examination. In selected cases, these biopsies were made in the operating room under anesthesia. At this moment, colonoscopy was performed in patients with initial obstructive tumors, as full endoscopic examination of the remaining large bowel was not possible at initial staging.

The presence of any significant residual ulcer or positive biopsies performed during proctoscopy was considered incomplete clinical response. Patients without any abnormality during tumor response assessment were considered to have complete clinical response.

Patients considered complete clinical responders during tumor response assessment were not immediately operated on. These patients were referred to monthly follow-up visits for repeat physical and digital rectal examination, proctoscopy, biopsies (when feasible), and serum CEA levels. Patients in this group were carefully advised that initial tumor remission could be temporary and, therefore, a strict follow-up adherence was mandatory. Abdominal and pelvic CT scans and chest radiographs were repeated every 6 months during the first year. Patients with sustained complete tumor regression for at least 12 months were considered stage 0(Observation group OB). During the second and third years after treatment, patients were advised to follow-up visits every 2 months and 6 months, respectively. Patients who developed distant metastasis at any time without recurrence of the primary tumor were treated exclusively for metastatic disease.

Lai 2016

Patients were assessed for their tumor response 8–12weeks after the completion of chemoradiotherapy with the same clinical and radiologic tools used in the baseline assessment of the tumor extent. All patients who were considered clinical complete responders according to rigorous criteria of clinical, endoscopic, and radiologic findings were treated without immediate radical surgery. The three criteria for cCR were (a) the absence of a residual ulceration, mass, or mucosal irregularity upon clinical/endoscopic assessment; (b) whitening of the mucosa and the presence of neovasculature (telangiectasia); and (c) radiologic imaging, such as CT, transrectal ultrasonography (TRUS), or MRI, without evidence of extrarectal residual disease, which were necessary for patients to be considered to have cCR. For example, lymph nodes <5 mm in their transverse diameter, or absence of irregular borders were considered negative for metastases, on standard T2-weighted MRI study or on CTscans. In addition, there was no evidence of hypoechoic, inhomogeneous lesion with irregular borders; thickening or destruction of the bowel wall under TRUS was also considered negative for extrarectal disease. The presence of clinical or endoscopic features of an incomplete response to CRTand the radiologic evidence of residual disease within the mesorectum were diagnostic of an incomplete clinical response, and therefore, radical surgery was recommended.

Experienced pathologists handled the pathologic evaluation of resected rectum specimen. The evaluation including (a) recording the length and the diameter of the rectum; (b) looking for and documenting the presence and appearance of any visible or palpable lesion and its location relative to the margins and to any landmarks; (c) dissecting the mesentery and thin section of the mesenteric fat, and examining and palpating each section for lymph nodes; and (d) submitting for histology the entire tumor site, each identified lesion, and lymph node. The percentage of pathological response ranged from no evidence of treatment effect (0 %) to complete regression with no viable tumor cells identified (100 %) and ypT0, corresponding to a percentage of pathological response of 100 %.

All patients with cCR were evaluated with outpatient visits every 3 months by a single experienced colorectal surgeon who performed a clinical examination in addition to a rigid proctoscopy or colonoscopy. Biopsy was utilizedselectively on any residual nodularity or scarring on exam. CEA levels were measured at the time of restaging (after finishing CRT at 8–12 weeks) and every 3 months thereafter. At the third year of follow-up, patients were examined every 6 months. The CEA cutoff value is ≤5 ng/dl at our institute, which has been demonstrated to have significant prognostic value in some studies. A radiologic imaging modality (including chest radiography, CT scans, and MRI) was used to exclude mesorectal disease and systemic status after 6 months and annually thereafter. CT scans were routinely obtained for all patients.

Lee 2015

MRI was performed approximately 6 weeks after the completion of nCRT. All magnetic resonance (MR) examinations were performed on a 3-Tesla MR scanner (Magnetom TrioTim; Siemens Medical Solutions, Erlangen, Germany) with multiple phased-array body coils. The imaging protocol included axial and oblique T1-weighted images and axial, sagittal, coronal, and oblique T2-weighted images. Axial diffusion-weighted MRI scans were also acquired. Detailed MRI techniques have been described previously. MRI-assessed tumor response was obtained by either of two experienced radiologists, particularly based on the diffusion-weighted MRI. Initial readings of MRI scans were utilized to define radiologic CR.

The patients were followed-up at 3-month intervals for 2 years, at 6-month intervals for the next 3 years, and annually thereafter. Follow-up examinations, including serum carcinoembryonic an-tigen tests, chest radiography or CT, abdominopelvic CT or MRI, colonoscopy, and positron emission tomography, were conducted on a semiannual basis or for suspected recurrences. The follow-up schedule was identical between the three groups. Disease-free survival (DFS) was measured from the date of completion of radiotherapy to the date of recurrence or death. LR-free survival (LRFS) was measured from the date of completion of radiotherapy to the date of LR or death. Recurrence was determined by clinical and radiological examinations or by histological confirmation.

Li 2015

Eight (26.7%) patients with a cCR received non-operative treatment because of religious reasons, fistula or poor physical condition, while twenty two (73.3%) patients were managed with a wait-and see-policy on the suggestion from clinicians because of the status of no any evidences of LR and/or DM. Patients in the surgery group prefer to receive surgery instead of the “wait-and- see” policy. The observation group was referred to monthly follow-up visits for digital rectal examination and measurement of serum CEA levels. Endoscopy with biopsy (as far as possible) and transrectal ultrasonography were performed every 3 months. In addition, abdominal and pelvic CT, MRI and chest radiography were repeated every 6 months beginning 1 year after completing NCRT. Patients in the observation group who sustained cCR for at least 1 year were referred to follow-up visits every 6 months during the second and third years after completing NCRT. After 3 years, follow-up visits were yearly. If any evidence of recurrence and/or metastasis was detected, salvage treatments, including radical surgery, local excision, radiotherapy with or without chemotherapy, or chemotherapy alone were carried out according to the situation of the patients.

Sections from all resected specimens were examined by local pathologists from five hospitals. The standardized protocol included determination of the AJCC TNM classification, stage grouping, number of examined and involved lymph nodes, presence or absence of lymphatic or venous invasion, tumor deposits and tumor regression grade (TRG). The reference pathologist tested pathological sections and then recorded the findings in a standardized document.

Mass 2011

The definition of a cCR is (1) substantial downsizing with no residual tumor or residual fibrosis only (with low signal on high value DWI, if available), shown in Figure 1. Residual wall thickening due to edema only was also an indication for a possible cCR; (2) no suspicious lymph nodes on MRI; (3) no residual tumor at endoscopy or only a small residual erythematous ulcer or scar; (4) negative biopsies from the scar, ulcer, or formertumorlocation;and(5)nopalpable tumor,wheninitially palpable with digital rectal examination. If patients did not meet all of these criteria, they were regarded as noncomplete responders.

An intensive follow-up protocol was incorporated into the wait-and see policy, consisting of digital rectal examination, MRI, endoscopy (with biopsy), computed tomography scan of the chest and abdomen (for distant metastasis imaging) and carcinoembryogenic antigen measurements. The follow-up schedule is provided in Appendix Table A1 (online only).

Renehan 2016

We used internationally recognised criteria to define clinical complete response, absence of residual ulceration, stenosis, or mass within the rectum during digital rectal examination and endoscopic examination 8 weeks or more after chemoradiotherapy completion. Classifi cation of clinical complete response required normal radiological imaging of the mesorectum and pelvis. For patients undergoing surgical resection, follow-up was in accordance with national guidelines. For patients managed by watch and wait, a more intensive follow-up protocol was used, consisting of outpatient digital rectal examination, MRI (every 4-6 months in the first 2 years), examination under anaesthesia or endoscopy, CT scan of the chest, abdomen, and pelvis, and at least two CEA measurements in the fi rst 2 years. We also determined the actuarial rate of local regrowth, using the date of the multidisciplinary team decision to watch and wait as the start time. We combined regrowths of mucosal lesions together with the less common submucosal or mesorectum-only regrowth lesions when we calculated regrowths.

Smith 2012

In all 32 NOM cases, a cCR was documented by (1) no palpable tumor on digital rectal examination and (2) endoscopy showing no visible pathology other than a flat scar. Patients were counseled that NOM is not standard treatment and might compromise oncologic outcome. Patients then commenced close follow-up at the discretion of the treating physicians, which generally entailed physical examinations and flexible sigmoidoscopies every 3 months for the first year and every 4 to 6 months thereafter. Use of diagnostic imaging was not standardized, but included cross-sectional imaging every 6 months for the first 2 years for most patients. Neither endorectal ultrasound nor rectal MRI was used routinely.

Yeom 2019

A cCR was defined as satisfying the following criteria, based on magnetic resonance imaging (MRI): (1) no residual tumor or residual fibrosis, and (2) no suspicious metastatic lymph nodes. The treatment approach and follow-up protocol were similar to our previous paper.

The patients who underwent RS were followed-up for 5 years at 6-month intervals, while the LE and WS groups were followed-up at 3-month intervals for 2 years and at 6-month intervals for the next 3 years. Follow-up examinations, including serum carcinoembryonic antigen, chest X ray or computed tomography and endoscopy, were conducted on a semiannual basis and MRI and positron emission tomography were added when recurrence was suspected, in accordance with according the National Comprehensive Cancer Network guidelines.

Wang 2020

1. no residual tumor or residual fibrosis was in digital rectal examination; (2) Typical performance of cCR was observed under endoscopy, such as white scars under the mucosa, new capillaries or normal intestinal wall, no ulcers or indurations, multi-point biopsy pathological results was negative (3) MRI showed that the original tumor site was normal intestinal wall or significant fibrosis; (4) CEA levels were completely normal; (5) CT examination indicated no distant metastasis.

The patients who underwent RS were followed-up for 5 years at 3-month intervals. Follow-up examinations, including serum carcinoembryonic antigen, chest X ray or computed tomography and endoscopy, were conducted on a semiannual basis and MRI and positron emission tomography
