# Supplementary material for: Efficacy and safety of wait and see strategy versus radical surgery and local excision for rectal cancer with cCR response after neoadjuvant chemoradiotherapy: a meta-analysis
Source: World J Surg Oncol. 2020 Aug 31;18:232. doi: 10.1186/s12957-020-02003-6 (PMC7457353; doi:10.1186/s12957-020-02003-6)
Supplement: Supplementary file 3 — Additional file 3:. The details of PICOS [file 12957_2020_2003_MOESM3_ESM.doc]

The details of PICOS are in additional file 3.

|  | The details of PICOS |
| --- | --- |
| P (Population) | Rectal cancer patients with cCR response after neoadjuvant chemoradiotherapy |
| I (Intervention ) | Wait and see strategy |
| C (Comparator) | Radical surgery  Local excision |
| O (Outcomes) | Primary objectives:  Local recurrence(LR): endoscopic pathology or other examination to confirm that the tumor that has regressed reappears  Distant metastasis(DM): the absence of metastases diagnosed by CT pathology or other methods  Cancer related death(CRD): The patient is related to death caused by cancer  Secondary objectives:  Disease-free survival(DFS): the absence of local or distant recurrence and death from any cause  Overall survival(OS) the absence of death from any cause |
