# Supplementary material for: Efficacy and safety of wait and see strategy versus radical surgery and local excision for rectal cancer with cCR response after neoadjuvant chemoradiotherapy: a meta-analysis
Source: World J Surg Oncol. 2020 Aug 31;18:232. doi: 10.1186/s12957-020-02003-6 (PMC7457353; doi:10.1186/s12957-020-02003-6)
Supplement: Supplementary file 4 — Additional file 4:. [file 12957_2020_2003_MOESM4_ESM.doc]

| Study | Mean tumor size(cm) | | | Mean distance from anal verge (cm) | | | Diabetes mellitus | | | Hypertension | | | Adjuvant chemotherapy  (perform,n) | | |
| --- | --- | --- | --- | --- | --- | --- | --- | --- | --- | --- | --- | --- | --- | --- | --- |
|  | WS | RS | LE | WS | RS | LE | WS | RS | LE | WS | RS | LE | WS | RS | LE |
| Ayloor[17] | - | - | - | 3 | 4 | - | - | - | - | - | - | - | - | - | - |
| Dalton[18] | 5.5 | 6.1 | - | 4.6 | 5.5 | - | - | - | - | - | - | - | - | - | - |
| Habr[11] | 3.6 | 4.2 | - | 3.6 | 3.8 | - | - | - | - | - | - | - | - | - | - |
| Lai[19] | - | - | - | 3.3 | 4.8 | - | - | - | - | - | - | - | - | - | - |
| Lee[20] | - | - | - | 2 | 4 | 2 | 1 | 4 | 4 | 2 | 8 | 6 | 1 | 23 | 12 |
| Li[21] | - | - | - | 3.5 | 3.8 | - | - | - | - | - | - | - | - | - | - |
| Mass[22] | - | - | - | 2.8 | 3.3 | - | - | - | - | - | - | - | 16 | 14 | - |
| Renehan[23] | - | - | - | 5 | 6 | - | - | - | - | - | - | - | - | - | - |
| Smith[24] | - | - | - | 6 | 7 | - | - | - | - | - | - | - | - | - | - |
| Yeom[25] | - | - | - | 4.2 | 4.6 | 3.5 | - | - | - | - | - | - | 5 | 124 | 18 |
| Wang[26] | - | - | - | - | - | - | - | - | - | - | - | - | - | - | - |

| Study | Radical surgery type | | | | | Pre-CRT CEA(ng/ml) | | | Post-CRT CEA(ng/ml) | | | BMI | | |
| --- | --- | --- | --- | --- | --- | --- | --- | --- | --- | --- | --- | --- | --- | --- |
|  | APR | LAR | LAR+CAA | Hartmann | Other | WS | RS | LE | WS | RS | LE | WS | RS | LE |
| Ayloor[17] | 9 | 1 | - | - | - | - | - | - | - | - | - | - | - | - |
| Dalton[18] | - | - | - | - | - | - | - | - | - | - | - | - | - | - |
| Habr[11] | 9 | 6 | 7 | - | - | - | - | - | - | - | - | - | - | - |
| Lai[19] | - | - | - | - | - | - | - | - | - | - | - | - | - | - |
| Lee[20] | 9 | 2 | - | - | 17 | 3.1 | 3.8 | 2.0 | 2.5 | 2.4 | 2.0 | - | - | - |
| Li[21] | 40 | 30 | 22 | - | - | - | - | - | - | - | - | - | - | - |
| Mass[22] | 6 | 14 | - | - | - | - | - | - | - | - | - | - | - | - |
| Renehan[23] | - | - |  | - | - | 3.0 | 3.0 | - | - | - | - | 26.5 | 25.8 | - |
| Smith[24] | - | - | - | - | - | - | - | - | - | - | - | - | - | - |
| Yeom[25] | - | - | - | - | - | 2.9 | 3.0 | 4.3 | 2.6 | 1.9 | 2.3 | - | - | - |
| Wang[26] | 47 | 132 | - | - | - | 2.7 | 3.5 | - | - | - | - | - | - | - |

CAA:coloanal anastomosis
