# Supplementary material for: Efficacy and safety of wait and see strategy versus radical surgery and local excision for rectal cancer with cCR response after neoadjuvant chemoradiotherapy: a meta-analysis
Source: World J Surg Oncol. 2020 Aug 31;18:232. doi: 10.1186/s12957-020-02003-6 (PMC7457353; doi:10.1186/s12957-020-02003-6)
Supplement: Supplementary file 5 — Additional file 5:. The details of neoadjuvant treatment of studies [file 12957_2020_2003_MOESM5_ESM.doc]

The details of neoadjuvant treatment of studies

| Study | Neoadjuvant therapy | PCR(%) |
| --- | --- | --- |
| Ayloor[17] | NCRT (Neoadjuvant chemoradiation). The specific plan is unknown. The assessment is undertaken at 4-6 weeks to evaluate the tumor. | 30 |
| Dalton[18] | 45 Gy in 25 fractions over 5 weeks with concurrent Capecitabine (825 mg⁄m^2^). A repeat MRI scan is undertaken at 6 to 8 weeks to evaluate the tumor. | 16.6 |
| Habr[11] | 50.40 Gy delivered at the isodose line given at 180 cGy/d for 5 days per week, for 6 consecutive weeks, using a 6-mV to 18-mV linear accelerator. 5-fluoracil (425 mg/m^2^/d) and folinic acid (20 mg/m^2^/d) administered intravenously for 3 consecutive days on the first and last 3 days of radiation therapy. The assessment is undertaken at 8 weeks to evaluate the tumor. | 8.3 |
| Lai[19] | 5-FU was administered as a bolus (350 mg/m^2^/day) with a low-dose leucovorin bolus (10 mg/m^2^/day) for 5 days on days 1–5 and 29–33 in combination with radiotherapy (45 Gy in 25 fractions or 54 Gy in 30 fractions). The assessment is undertaken at 8-12 weeks to evaluate the tumor. | - |
| Lee[20] | 5-fluorouracil-based chemotherapy with concomitant long course radiation (50.40 Gy in 28 fractions). The specific plan is unknown. The assessment is undertaken at 6-10 weeks to evaluate the tumor. | 42.9 |
| Li[21] | NCRT (50 Gy/25 f/2 Gy, capecitabine, 825 mg/m^2^ bid, concurrently). The assessment is undertaken at 8-10 weeks to evaluate the tumor. | 88 |
| Mass[22] | CRT consisted of 28 fractions of 1.8 Gy combined with 2*825 mg/m^2^ capecitabine. The assessment is undertaken at 6-8 weeks to evaluate the tumor. | - |
| Renehan[23] | 45 Gy in 25 daily fractions with concurrent fluoropyrimidine-based chemotherapy for 34 days. The specific plan is unknown. The assessment is undertaken at 8 weeks to evaluate the tumor. | - |
| Smith[24] | The median dose of radiotherapy administered was 50.40 Gy (range 45–56 Gy). plus 5-flfluorouracil (5-FU) or capecitabine. The specific plan is unknown. The assessment is undertaken at 4-10 weeks to evaluate the tumor. | - |
| Yeom[25] | Long-course radiation (50.40 Gy in 28 fractions) with capecitabine, Capeox or 5-FU. The specific plan is unknown. The assessment is undertaken at 8 weeks to evaluate the tumor. | 33.9 |
| Wang[26] | CRT consisted of GTV 50 Gy/25 fractions /5 weeks, CTV 45-46 Gy/ fractions /5 weeks combined with 2-4 cycles 5-fluorouracil or capecitabin based chemotherapy. The specific plan is unknown. The assessment is undertaken at 6-12 weeks to evaluate the tumor. | - |

| Study | 2-year OS (%) | | | 2-year DFS (%) | | | 5-year OS (%) | | | 5-year DFS (%) | | |
| --- | --- | --- | --- | --- | --- | --- | --- | --- | --- | --- | --- | --- |
|  | WS | RS | LE | WS | RS | LE | WS | RS | LE | WS | RS | LE |
| Ayloor[17] | - | - | - | - | - | - | - | - | - | - | - | - |
| Dalton[18] | 100 | 100 | - | 100 | 100 | - | - | - | - | - | - | - |
| Habr[11] | 100 | 90.9 | - | 98.5 | 86.3 | - | 100 | 90.9 | - | 95.7 | 86.3 | - |
| Lai[19] | 100 | 100 | - | - | - | - | 100 | 92.3 | - | - | - | - |
| Lee[20] | - | - | - | 75 | 89.2 | 62.5 | - | - | - | - | - | - |
| Li[21] | 100 | 100 | - | 96.6 | 98.9 | - | 100 | 95.6 | - | 90 | 92.3 | - |
| Mass[22] | 100 | 95 | - | 90.4 | 95 | - | - | - | - | - | - | - |
| Renehan[23] | 82.9 | 91.7 | - | 77.5 | 81.6 | - | - | - | - | - | - | - |
| Smith[24] | 96.8 | 100 | - | 87.5 | 98.2 | - | - | - | - | - | - | - |
| Yeom[25] | - | - | - | - | - | - | 20 | 99.2 | - | 20 | 85.2 | - |
| Wang[26] | - | - | - | - | - | - | 89.8 | 97.7 | - | - | - | - |
