# Supplementary material for: Efficacy and safety of wait and see strategy versus radical surgery and local excision for rectal cancer with cCR response after neoadjuvant chemoradiotherapy: a meta-analysis
Source: World J Surg Oncol. 2020 Aug 31;18:232. doi: 10.1186/s12957-020-02003-6 (PMC7457353; doi:10.1186/s12957-020-02003-6)
Supplement: Supplementary file 6 — Additional file 6:. The details of Inclusion and Exclusion Criteria [file 12957_2020_2003_MOESM6_ESM.doc]

The details of Inclusion and Exclusion Criteria are in additional file 6.

|  | The details of inclusion and exclusion Criteria |
| --- | --- |
| Inclusion Criteria | 1.pathological and long-term outcomes were compared between wait and see versus radical surgery or local excision for rectal cancer with cCR response after neoadjuvant chemoradiotherapy;  2.surgery included radical surgery and local excision;  3.cCR response after neoadjuvant chemoradiotherapy(criteria for ccR were mentioned above) [9];  4.RCT(Randomized Controlled Trial), RCNTs(retrospective comparative  non-randomizedstudies), PCNTs(prospective comparative non-randomized  studies), cohort studies or case-control studies. |
| Exclusion Criteria | 1. studies with no valuable outcome;  2. patients were not well grouped or confusing group was not suitable for the purpose of the article;  3. bad clinical response of rectal cancer after neoadjuvant chemoradiotherapy |
