# Supplementary figures and images for: Efficacy and safety of wait and see strategy versus radical surgery and local excision for rectal cancer with cCR response after neoadjuvant chemoradiotherapy: a meta-analysis
Source: World J Surg Oncol. 2020 Aug 31;18:232. doi: 10.1186/s12957-020-02003-6 (PMC7457353; doi:10.1186/s12957-020-02003-6)

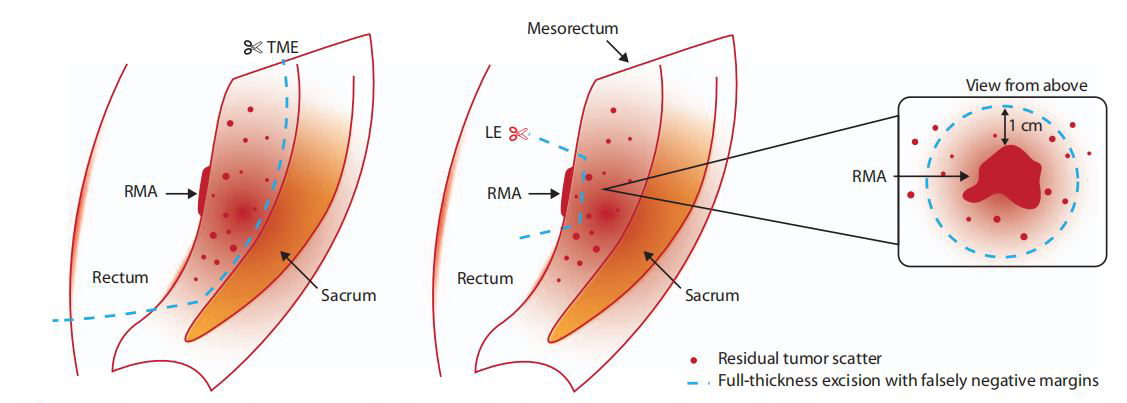

Supplement: Supplementary file 8 — Additional file 8:. [file 12957_2020_2003_MOESM8_ESM.tif]
